# Patient satisfaction, needs, and preferences concerning information dispensation at the emergency department: a cross-sectional observational study

**DOI:** 10.1186/s12245-022-00407-7

**Published:** 2022-01-24

**Authors:** Marank de Steenwinkel, Juanita A. Haagsma, Esther C. M. van Berkel, Lotte Rozema, Pleunie P. M. Rood, Marna G. Bouwhuis

**Affiliations:** 1 Department of Emergency Medicine, Erasmus University Medical Center Rotterdam, Room Nc-017, P.O. Box 2040, 3000 CA Rotterdam, The Netherlands; 2 Department of Public Health, Erasmus University Medical Center Rotterdam, Rotterdam, The Netherlands

**Keywords:** Information dispensation, Patient satisfaction, Emergency department, Waiting times

## Abstract

**Background:**

Patient satisfaction is an important indicator of emergency care quality and has been associated with information dispensation at the emergency department (ED). Optimal information dispensation could improve patient experience and expectations. Knowing what kind of information patients want to receive and the preferred way of information dispensation are essential to optimize information delivery at the ED. The purpose of this cross-sectional observational study was to evaluate patient satisfaction concerning information dispensation (including general, medical, and practical information), the need for additional information, and preferences with regard to the way of information dispensation at the ED of a teaching hospital in the Netherlands.

**Results:**

Four hundred twenty-three patients (patients ≥ 18 years with Glasgow Coma Scale 15) were enrolled (response rate 79%). The median patient satisfaction score concerning the overall information dispensation at the ED was 7.5 on a rating scale 0–10. Shorter length of ED stay was associated with higher patient satisfaction in multivariate analysis (*P* < 0.001). The majority of respondents were satisfied regarding medical (*n* = 328; 78%) and general information (*n* = 233; 55%). Patients were less satisfied regarding practical information (*n* = 180; 43%). Respondents who indicated that they received general, medical and practical information were significantly more often satisfied compared to patients who did not receive this information (*P* < 0.001). Two thirds (*n* = 260; 62%) requested more general information. Half of the respondents (*n* = 202; 48%) requested more practical information and a third (*n* = 152; 36%) requested more medical information. The preferred way for receiving information was orally (*n* = 189; 44.7%) or by leaflets (*n* = 108; 25.5%).

**Conclusion:**

The majority of respondents were satisfied concerning information dispensation at the ED, especially regarding medical information. Respondents requested more general and practical information and preferred to receive the information orally or by leaflets.

## Background

Patient satisfaction is one of the important indicators of emergency care quality, outcomes of health care services and patient treatment adherences [1–3]. Proper information dispensation has been associated with patient satisfaction [4]. Patients who received additional information were more satisfied at the emergency department (ED) [3, 5–9]. Welch et al. showed that a lack of information about progress and delays had a greater effect on patient satisfaction than perceived waiting times [5]. These studies evaluated overall patient satisfaction, but did not focus on patient satisfaction concerning information dispensation during the ED visit.

Optimal information dispensation could improve patient experience and expectations at the ED. Improvement of information delivery requires a patient-centered approach, meaning information that is respectful and responsive to individual patient preferences, needs, and values. Therefore, knowing what kind of information the patient wants to receive is essential. Only a few studies described that patients wanted to receive more general information (e.g., waiting times, triage, identifying staff, the progress during an ED visit) or practical information (e.g., parking, Wi-Fi, or about common medical emergencies) [10–12]. These studies were all conducted in non-European hospitals. However, information concerning patients’ needs for receiving medical information during the ED visit (e.g., information regarding invasive and non-invasive procedures or medication), is currently lacking.

There are a growing number of studies in the ED population introducing different ways of information dispensation, including more modern techniques such as videos, websites, and apps [8, 13–15]. Nevertheless, there are only a few non-European studies regarding the preferred way of information dispensation at the ED. These studies showed that patients preferred information dispensation by video, leaflets, or information screen in the waiting room [11, 12, 16].

In order to optimize patient information dispensation during the ED visit, the primary objective was to evaluate patient satisfaction concerning overall patient information. The secondary objectives included patient satisfaction concerning the delivery of general, medical, and practical information, the needs, and preferred way of information dispensation in the ED population.

## Methods

### Study design and procedures

This study was a cross-sectional observational single center study at the ED of the Erasmus MC Rotterdam. The Erasmus MC is an urban university teaching hospital with approximately 26,000 ED visits per year. Ethical approval was obtained from the research ethics board before start of the study (MEC-2018-1577).

During the study period, 16 January 2019 to 10 March 2019, all patients ≥ 18 years who visited the ED with a Glasgow Coma Scale 15 were eligible for inclusion. Patients were approached for informed consent after completing treatment at the ED before discharge or admission to hospital to prevent information bias. Exclusion criteria included no informed consent, not able to understand Dutch or red-triaged patients (Manchester Triage System) or who were considered too ill by attending nurses and the emergency physician. Patients were recruited with equal distribution during different days of the week and different shifts to ensure a good reflection of the ED population. Initially, patients were also recruited during night shifts. However, only a few patients were eligible for recruitment during these shifts and therefore recruitment was only continued during day- and evening shifts. After obtaining informed consent a written questionnaire was distributed by an independent researcher. Patients independently filled out the survey to prevent biases by attending physicians or nurses. The questionnaire was handled anonymously. Patients could leave the study at any time without any consequences. There was no follow-up in this study.

### Questionnaire

The questionnaire was developed in Dutch by emergency physicians and an epidemiologist and was validated for face and content validity. For this purpose, 4 selected experts in patient education, 10 medical professionals (e.g., emergency nurses, emergency physicians or residents), 10 laypersons, and 10 patients were asked to review the questionnaire. The results were used to improve the questionnaire. The questionnaire consisted of questions about patient satisfaction, needs and preferences regarding patient information delivery at the ED. The questionnaire contained questions about patient characteristics (age, sex, highest attained education, migration background, and previous ED visits in Erasmus MC) and patient satisfaction regarding information dispensation. Patient satisfaction was subdivided in satisfaction concerning overall information dispensation grading on a rating scale of 0–10 (0 extremely dissatisfied–10 extremely satisfied) and satisfaction regarding general, medical and practical information delivery grading on a 5-point Likert scale (very unsatisfied–very satisfied). Additionally, patients were asked to indicate whether or not they received additional information. General information included information about logistics of the ED visit; waiting times, triage, identification and function hospital staff, privacy, and costs. Medical information represented information about non-invasive procedures (electrocardiogram, X-ray), invasive procedures (blood samples, intravenous system, computed tomography scan, epidural), medication (pain-, provided - and discharge medication), information regarding monitoring vital parameters of the patients and access to patient files. Practical information included information about parking, restrooms, Wi-Fi, food and drinks, taxi/public transport, and pharmacy availability.

The questionnaire also included questions regarding patients’ needs for additional information regarding general, medical, and practical information. Patients were able to give multiple answers.

Finally, patients were asked about their preferred way of information delivery at the ED subdivided in orally, leaflets, poster, video, website, and mobile app. Multiple answers were allowed.

### Primary and secondary outcomes

The primary outcome of this study was the patient satisfaction score concerning the overall patient information dispensation. Secondary outcomes included patient satisfaction concerning the delivery of general, medical and practical information, needs, and preferred way of information delivery during the ED visit in percentages.

### Statistical analysis

The responses on the completed questionnaires were collected into a database in IBM SPSS Statistics version 25. This database was supplemented by baseline characteristics (day and shift of ED visit, self-referral or referral by medical specialist or general practitioner, arrival by own transport or ambulance, triage category, length of stay of the ED visit, and destination after ED visit) from the electronic patient files. Uncompleted questions were coded as missing data. The baseline characteristics and secondary outcomes have been analyzed by descriptive statistics given as average including 95% confidence intervals (95% CI). The primary outcome has been given as median including 95% CI.

Based on an estimated eligible study population of 17,846 patients yearly (patients under 18 years, red-triaged and 50% of the orange-triaged adult patients excluded because of Glasgow Coma Scale < 15), with margins of error of 5% and a confidence interval of 95%, a representative sample size of 377 patients was calculated. Mann-Whitney *U* tests were conducted to test for differences in patient satisfaction concerning overall patient information dispensation in patients who did and did not receive information. Uni- and multivariate analyses were used to determine associations between patient characteristics and patient satisfaction score concerning overall patient information dispensation. Patient characteristics with a significance less than 0.05 were applied for multivariate logistic linear forward regression analysis. A statistically significant correlation was defined by *p* value less than 0.05.

## Results

**Fig. 1:**
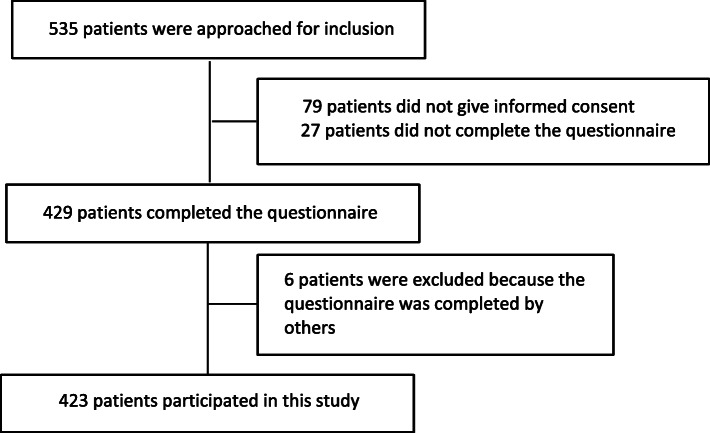
Patient enrollment

In this study 535 eligible patients were approached to participate of which 423 patients completed the questionnaire (response rate 79%) (Fig. 1). In total 251 patients (59%; 95% CI 55–64%) of the study population were male and mean age of the respondents was 53.7 years (SD ± 18.3). The majority (64%) of the respondents did not have a migration background. Approximately half (48%) of the study population had never visited the ED of the Erasmus MC before. 208 respondents (49%) were referred by a general practitioner or medical specialist. The baseline characteristics are summarized in Table 1.

**Table 1:** Baseline characteristics of the study population

Variable	*n* (%) – *N* = 423	95% Confidence interval
Age, mean in years	53.7	51.7–55.3
Sex		
Male Female	251 (59.3) 172 (40.7)	0.55–0.64 0.36–0.46
Education^*^		
Low Middle High Missing	159 (37.6) 124 (29.3) 133 (31.4) 7 (1.7)	0.33–0.42 0.25–0.34 0.27–0.36
Migration background^**^		
Native Dutch No native Dutch Missing	272 (64.3) 145 (34.3) 6 (1.4)	0.60–0.69 0.30–0.39
Previous ED visits in last 3 years		
0 1–2 ≥ 3 Missing	201 (47.5) 121 (28.6) 98 (23.2) 3 (0.7)	0.43–0.52 0.25–0.33 0.19–0.27
Referral		
Self-referral Referral by general practitioner/specialist	215 (50.8) 208 (49.2)	0.46–0.56 0.44–0.54
Arrival		
Own transportation Ambulance	306 (72.3) 117 (27.7)	0.68–0.76 0.24–0.32
Shift		
Day Evening Night	241 (57.0) 146 (34.5) 36 (8.5)	0.52–0.62 0.30–0.39 0.06–0.12
Day		
Monday–Friday Weekend	313 (74.0) 110 (26.0)	0.70–0.78 0.22–0.30
Triage category		
Orange Yellow Green Missing	61 (14.4) 262 (61.9) 98 (23.2) 2 (0.4)	0.11–0.18 0.57–0.66 0.19–0.27
Destination after ED visit		
Discharged Admission Transfer to other hospital Left against medical advice	265 (62.6) 130 (30.7) 26 (6.1) 2 (0.5)	0.58–0.67 0.26–0.35 0.04–0.09
Length of stay ED visit, mean in minutes	253	253–271

^*^Low education no education, pre-primary, primary, lower secondary education, compulsory education. *Middle education* upper secondary general education, basic vocational education, secondary vocational education, post-secondary education, *High education* specialized vocational education, university/college education, (post-) doctorate, and equivalent degrees

^**^Native Dutch both parents were born in the Netherlands. *No Native Dutch* one or both parents were not born in the Netherlands

### Primary outcome

The median patient satisfaction score concerning overall patient information delivery was 7.5 (95% CI 7.13–7.47). A total of 224 respondents (77%) scored patient information dispensation 7 or higher in contrast to 53 respondents (13%) who scored 5 or lower.

Univariate linear logistic regression analysis showed that referral by medical specialist or general practitioner (95% CI − 0.671 to −0.011; *P* = 0.04) and longer length of ED visit (95% CI − 0.003 to − 0.001; *P* < 0.001) were significantly associated with a lower patient satisfaction score concerning overall patient information delivery. In multivariate analysis only longer length of ED visit was significantly associated with lower patient satisfaction concerning overall information dispensation (95% CI − 0.003 to − 0.001; *P* < 0.001).

### Secondary outcomes

#### Patient satisfaction concerning general, medical, and practical information dispensation

**Fig. 2:**
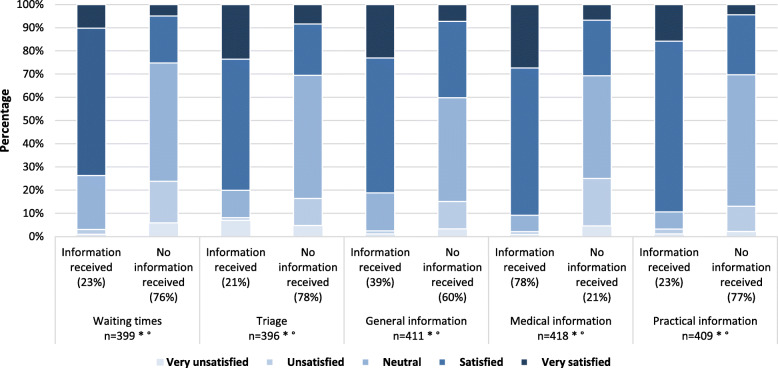
Patient satisfaction concerning general, medical, and practical information dispensation. Patient satisfaction concerning waiting times, triage, general (e.g., logistics, costs), medical (e.g., medical procedures), and practical (e.g., Wi-Fi, food and drinks) information dispensation at the ED on a 5-point Likert scale. The results in each information dispensation group were subdivided into patients who indicated that they received information vs. patients who indicated that they did not receive information. * Missing data: waiting times *n* = 27, triage *n* = 24, general information *n* = 12, medical information *n* = 5, practical information *n* = 14. °Respondents who indicated that they received information concerning triage (*P* < 0.001), waiting times (*P* < 0.001), and general (*P* < 0.001), medical (*P* < 0.001), or practical information (*P* < 0.001) were significantly more satisfied compared to patients who did not received that information

The majority of respondents were satisfied or very satisfied, on a 5-point Likert scale, regarding medical information (*n* = 328; 78%) and general information (*n* = 233; 55%). Patients were less satisfied concerning triage (*n* = 166; 39% of respondents satisfied or very satisfied), waiting times (*n* = 151; 36% of respondents satisfied or very satisfied), and practical information (*n* = 180; 43% of respondents satisfied or very satisfied). Respondents who indicated that they received information concerning triage, waiting times and general, medical or practical information were significantly more satisfied compared to patients who did not received that information (*P* < 0.001) (Fig. 2).

#### Patients’ needs concerning information dispensation

The results are summarized in Table 2**.** Of 423 respondents, 260 respondents (62%) wanted more general information, of which the most frequently registered needs were information concerning waiting times (*n* = 194; 75%) and triage (*n* = 110; 42%). In univariate linear logistic regression analysis lower age (OR 0.99; 95% CI 0.975–0.997; *P* = 0.01) and migration background (OR 0.50; 95% CI 0.32–0.77; *P* = 0.002) were significantly associated with a greater need for general information.

**Table 2:** Patients’ needs with regards to general, medical and practical information

Variable	*n* (%)^* ^– *N* = 423	95% Confidence interval
General information° Waiting times Triage Name/function medical staff	260 (61.5) 194 (74.6) 110 (42.3) 54 (20.8)	0.57–0.66 0.69–0.80 0.36–0.48 0.16–0.26
Medical Information° Invasive procedure^**^ Medication Non-invasive procedure^***^	152 (35.9) 87 (57.2) 79 (52.0) 63 (41.4)	0.32–0.41 0.49–0.65 0.44–0.60 0.34–0.50
Practical information° Food and drinks Parking Wi-Fi	202 (47.8) 103 (51.0) 88 (43.6) 66 (32.7)	0.43–0.53 0.44–0.58 0.37–0.51 0.27–0.39

°Missing total study population: general information 7 (1.7%), medical information 18 (4.3%), practical information 11 (2.6%)

^*^Patients had the option to choose more answers for desires in the general, medical and practical information group. For that reason, the percentages are not equal to 100%.

^**^Invasive procedure: blood sample, iv system, CT, and epidural

^***^Non-invasive procedure: X-ray and electrocardiogram

In regard to medical and practical information, respectively 152 respondents (36%) and 202 respondents (48%) indicated a need for more information. Regarding medical information respondents wanted to receive more information about invasive procedures (*n* = 87; 57%), medication (*n* = 79; 52%), and non-invasive procedures (*n* = 63; 41%). In univariate analysis, migration background was significantly associated with increasing need for medical information (OR 0.46; 95% CI 0.30–0.70; *P* < 0.01). Concerning practical information 103 respondents (51%) requested more information about food and drinks, followed by 88 respondents (44%) about parking and 66 respondents (33%) about Wi-Fi.

#### Preferred way of information dispensation

The preferred way for receiving patient information was orally (*n* = 189; 44.7%) or by leaflets (*n* = 108; 25.5%) (Table 3). Seven respondents did not indicate their preferences in information dispensation. The less preferred way of information delivery was by website (*n* = 36; 8.5%) and poster (*n* = 20; 4.7%). Univariate analysis showed that lower age (OR 0.98; 95% CI 0.974–0.995; *P* = 0.004) was significantly associated with the preference for oral information delivery. Migration background (OR 0.60; 95% CI 0.38–0.94; *P* = 0.03) and increased length of stay at the ED (OR 1.001; 95% CI 1.000–1.002; *P* = 0.04) were significantly associated with the preference for information delivery by leaflets.

**Table 3:** Patient preferences in the way of information delivery

Variable	*n* (%)^* ^– *N* = 423	95% Confidence interval
Oral information	189 (44.7)	0.40–0.49
Leaflets	108 (25.5)	0.22–0.30
App Video Website Poster No preferences indicated	76 (18.0) 69 (16.3) 36 (8.5) 20 (4.7) 7 (1.7)	0.15–0.22 0.13–0.20 0.06–0.12 0.03–0.07 0.01–0.03

^*^Patients were able to choose more answers for preferences in the way of information delivery

## Discussion

Our study shows that respondents were satisfied concerning patient information dispensation at the ED. Respondents who indicated that they received information concerning general, medical, and practical information were more satisfied. A longer length of stay at the ED was associated with a lower patient satisfaction concerning overall patient information delivery. Respondents indicated that they would like to receive more general information, especially about waiting times and triage. Younger age and migration background were associated with increased needs for information. The preferred way of receiving patient information was orally in younger respondents or by leaflets in respondents with a longer length of ED stay or migration background.

This is the first study that investigated patient satisfaction concerning overall information dispensation at the ED. In multivariate analyses, longer length of the ED visit was associated with a lower patient satisfaction score concerning overall patient information delivery, which is consistent with studies regarding overall patient satisfaction concerning the ED visit [9, 17]. Additionally, the most frequently requested information concerned waiting times (75%), which is in line with 14–75% reported in previous studies [10–12]. Information dispensation regarding waiting times was associated with a higher overall patient satisfaction [1, 3, 7, 17–19]. Seibert et al. and Alhabadan et al. showed that respondents preferred an update concerning waiting times every 41 min and 30 min respectively [11, 12].

In this study, the need for general information dispensation at the ED was associated with lower age, which was also observed in a systematic review in the German population [20]. Likewise, patients with a migration background requested more frequently general and medical information. Although this was previously described, it remains unclear why patients with a migration background prefer to receive more information during the ED visit [10, 12]. The current study does not answer this question. Nevertheless, it is important to take age and migration background into account when optimizing patient information dispensation during the ED visit.

Recent literature showed multiple studies introducing different ways of information dispensation at the ED [8, 13–15]. However, the preferred way of information dispensation at the ED was only based on non-European studies with varying results. The preferred way of information delivery was by leaflets (32–60%), video (25–50%) or speaking with an expert (24%) [11, 12, 16]. The option for modern techniques such as apps on personal devices or tablets provided by the hospital was not studied before. Interestingly, this study did not show preferences for modern techniques with half of the respondents preferring to receive the information orally.

There were multiple strengths to this study. The first strength was the high response rate leading to a representative reflection of the ED population at the Erasmus MC. Also more patients were enrolled than the sample size calculation of 377 patients to mitigate unexpected missing data. Furthermore, patients were included after completing treatment at the ED before discharge or admission to hospital to prevent information bias. Thirdly, the developed questionnaire concerned many questions about patient characteristics and many aspects of patient satisfaction, needs and preferences regarding patient information delivery at the ED. This allowed us to determine which patient characteristics were associated with the different aspects of patient satisfaction, needs, and preferences regarding patient information dispensation. Finally, face and content validity was applied to optimize the non-validated questionnaire by feedback from experts, medical staff, patients, and laypersons.

There are limitations to this study. The first limitation was the single center study design. The Erasmus MC is an academic urban hospital with certain patient characteristics; therefore, the results could be less applicable to rural hospitals. Secondly, due to differences in health care system between the Netherlands and other countries, the results of this study may be less applicable to other countries. Lastly, there were missing data concerning the secondary outcomes, which could have introduced potential bias and reduced generalizability of the results.

Based on the study results, we recommend that in daily practice attention must be paid to patient information dispensation during the ED visit, especially regarding waiting times, triage, and food and drinks. The preferred way of information dispensation is person dependent and might change over time. At this time, implementation of more general and practical information by leaflets could be a good and low-cost improvement. Nevertheless, when implementing modern techniques, this should be evaluated to ensure that it meets the expectations of the ED population.

## Conclusions

This study showed that respondents were satisfied with overall information dispensation during the ED visit. However, there was a need for more patient information regarding general and practical information. The preferred method to receive the information was orally or by leaflets.

## Data Availability

The datasets used and/or analyzed during the current study are available from the corresponding author on reasonable request.
